# Retinal Plasticity

**DOI:** 10.3390/ijms23031138

**Published:** 2022-01-20

**Authors:** Enrica Strettoi, Beatrice Di Marco, Noemi Orsini, Debora Napoli

**Affiliations:** 1 CNR Neuroscience Institute, 56124 Pisa, Italy; beatrice.dimarco@in.cnr.it (B.D.M.); noemi.orsini@in.cnr.it (N.O.); debora.napoli@in.cnr.it (D.N.); 2 Regional Doctorate School in Neuroscience, Universities of Florence, Pisa and Siena, 50134 Florence, Italy

**Keywords:** retinitis pigmentosa, remodeling, structural plasticity, deafferentation

## Abstract

Brain plasticity is a well-established concept designating the ability of central nervous system (CNS) neurons to rearrange as a result of learning, when adapting to changeable environmental conditions or else while reacting to injurious factors. As a part of the CNS, the retina has been repeatedly probed for its possible ability to respond plastically to a variably altered environment or to pathological insults. However, numerous studies support the conclusion that the retina, outside the developmental stage, is endowed with only limited plasticity, exhibiting, instead, a remarkable ability to maintain a stable architectural and functional organization. Reviewed here are representative examples of hippocampal and cortical paradigms of plasticity and of retinal structural rearrangements found in organization and circuitry following altered developmental conditions or occurrence of genetic diseases leading to neuronal degeneration. The variable rate of plastic changes found in mammalian retinal neurons in different circumstances is discussed, focusing on structural plasticity. The likely adaptive value of maintaining a low level of plasticity in an organ subserving a sensory modality that is dominant for the human species and that requires elevated fidelity is discussed.

## 1. Paradigms of Plasticity

Credit for using the term plasticity to designate modifications in nervous paths taking place with the formation of specific behaviors is given to William James, who used the word in his *Principles of Psychology*, published in 1890. While the name has been maintained successfully ever since, its present meaning has expanded to account for the neurobiological complexity and multifaceted nature of this remarkable brain property. Complexity arises from the fact that the brain is simultaneously the source of behavior and the receiver of the modifications that behavior produces, and this circular process constitutes the core of learning, memory and cognition [1]. 

The capability of being molded into various forms is proper of numerous organic, synthetic or processed materials that are therefore collectively designated as “plastic”; however, their modifiable nature is almost entirely passive, not at all like nervous plasticity, a highly active and energy-demanding process. 

The ability of the CNS to actively modify as a function of environmental inputs might take different forms [2,3,4,5,6]. The well-known Hebbian plasticity occurs in numerous brain areas and is induced by correlated spiking of neurons, whose discharge becomes consequently amplified. It can be induced on a timescale of seconds and might have long-lasting effects. Hebbian plasticity is therefore defined as the occurrence of synapse-specific changes in strength driven by the coordination of presynaptic input and postsynaptic firing; thus, neurons that fire together also wire together. Experimentally, Hebbian plasticity is achieved by strengthening (long-term potentiation (LTP)) or weakening (long-term depression (LTD)) specific excitatory synapses using different frequencies of electrical stimulation. High-frequency trains of stimulation of presynaptic neurons elicit potentiation of postsynaptic responses (LTP), meaning that cells become more sensitive to stimulation. On the contrary, low-frequency inputs induce milder depolarization, requiring stronger stimuli to generate spikes (LTD). The final consequence of Hebbian plasticity is an activity-dependent change of synaptic efficacy as a function of positive feedback; for these properties, it is typically linked to memory and learning [7]. 

Homeostatic plasticity, instead, acts to stabilize the activity of a neuron or neuronal network, adapting its output to compensate for variations (i.e., pathological loss of inputs caused by cell death). It is generally assumed to be adaptive and compensatory. Finally, structural plasticity refers to the CNS ability to continuously modify its fine physical structure, insofar as not only excitatory synapses modify the membrane expression of glutamate receptors modulating the activity of neuronal networks, but rewiring and selective stabilization of synapses also occur, forming the physical substrate of learning and memory processes [8].

As shown by theoretical and experimental neuroscience studies, the essence of plasticity lies in the ability of certain synapses to be actively modified by experience: both number and strength of synaptic contacts change drastically during development or in response to variable environmental stimuli, triggering, among other outcomes, readjustment of neuronal number and remodeling of dendritic and axonal arbors, creating memory traces throughout life [5]. 

## 2. The Endless Hippocampal Plasticity

Molecular processes changing synaptic strength to create memory traces underlie the well-known mechanisms of long-term depression and long-term potentiation (LTD and LTP) [6], mostly studied in the hippocampus, which is the designated place for laying down explicit memories and therefore expected to be modifiable. Hippocampal synapses are characterized by activity-dependent plasticity, and lesions of this brain area greatly limit the ability to gain new episodic memories. 

Plasticity can be regulated by multiple factors, including drugs, steroids, nutrients and nutrient-dependent hormones, such as insulin, whose modulatory effects on hippocampal LTP and LTD have been described in detail [9]. Modulators of plasticity are also positive and negative stressors, as well as sensorial and somatosensory experiences. Independently of the nature of regulators, neuronal plasticity in mammals is largely put into action through changes of synaptic contacts between pre-existing cells (synaptic formation, elimination and change in strength) [10]. Indeed, the hippocampus is endowed with a high degree of so-called structural plasticity [11], which influences the number of synapses and the formation of novel connections among neurons, as well as the complexity of their dendritic arborizations. While a rearrangement of these components is shared with other brain areas during development, it is mainly in the hippocampus that structural plasticity is maintained throughout life, and mature hippocampal neurons can undergo changes that induce dendritic spine modifications [12]. LTP can cause spine expansion in the order of seconds, while LTD causes spine shrinkage. In addition, hippocampal granule cells show the unique property to exhibit active neurogenesis, even in humans, at least until adolescence [13]. The precise mechanisms of structural plasticity and the effects on hippocampal physiology are yet to be completely clarified; in particular, the role of adult neurogenesis on hippocampal properties is still debated. However, the general idea emerging from comparative approaches is that an increment in brain size during evolution has led to an increased neuronal computation capacity which, in turn, has enforced a dropdown of neurogenesis in the whole CNS. An obvious drawback of this restriction is shown when brain repair is needed, e.g., in traumatic or neurodegenerative conditions, because, evidently, CNS stability and neurogenesis are not meant to coexist [14]. However, the neurogenic reserve endowed in the hippocampus supports the exceptionally high structural plasticity of this part of the brain, contributing to keeping it flexible and plastic. This favors learning when novelty is introduced in the environment. Thus, incorporation of even a few new neurons into the adult human hippocampus provides an advantage throughout an individual’s life and supports adaptation to new experiences [14,15]. 

Even in the hippocampus, however, the recovery potential due to plasticity shows restrictions. Traumatic or genetically driven loss of sensory modalities (providing to the hippocampus information fundamental for memory formation) results in major impairment of function. Mouse models of progressive, genetic hearing loss undergo a conspicuous cortical molecular and functional reorganization, which comprises altered expression of neurotransmitter receptors [16]. This is followed by major impairment in hippocampal LTP and spatial memory, indicating how any kind of sensorial depletion (i.e., associated with aging or vascular abnormalities) constitutes a risk factor favoring cognitive decline. Plasticity can only compensate for the impairment of information processing at the hippocampal level but not for large sensorial deficits.

## 3. Plasticity in the Visual Cortex: A Developmental Property

For the neocortex, experimental paradigms involving manipulation of the visual experience (lid suturing, dark rearing, and eye patching, applied to various species to reduce or unbalance sensorial input) constitute the mainstay of the literature on plasticity and have been used for decades to manipulate the physiology of visual neurons, studying the effects on binocular vision from organ level to molecular mechanisms. These studies have contributed to the definition of fundamental concepts, such as the existence of a critical period for the visual system, and the notion that the structure and function of cortical circuits are profoundly affected when alterations in experience occur. Critical periods, true windows of different time of onset and duration for different modalities [17], exist for many other functions, typically in the auditory system, in such areas as those controlling human language, and in all species, i.e., from monkeys to fruit flies [18].

The possibility to modify permanently the anatomy and physiology of the primary visual cortex has been clearly demonstrated by studying the effects of monocular deprivation, leading to the definition of ocular dominance plasticity (ODP), the reorganization of cortical neural circuits and incoming afferents in response to an unbalanced light stimulation [17,19,20].

In many mammals, neurons of the primary visual cortex (V1) are organized in alternating stripes called ocular dominance columns (ODCs) and respond preferentially to inputs received by the left or right eye (monocular neurons). Axonal projections from the dorsal lateral geniculate body (LGNd) create connections with cells in layer 4 of V1. Spontaneous, nonsynchronous activity originating from the retina [21] and traveling through the thalamus and the cortex before eye opening is the major factor responsible for the proper organization of such neuronal architecture [22,23]. Indeed, ocular dominance columns do not develop normally if spontaneous activity is blocked, showing that instructions carried over during this postnatal phase are necessary for the proper topographical organization of visual afferents [24,25]. When eyes open, light stimuli replace spontaneous activity; however, in the case of unmatched inputs from the two eyes early in life, the normal organization of thalamic–cortical afferents is profoundly reorganized. The deprived eye loses a large set of connections in favor of the other one, which takes over, to the point that its connections with the visual cortex become anatomically and functionally predominant. Cortical projections connected to the favorite eye enlarge their territories at the expense of the others, causing a macroscopic resizing of the ODCs, which expand in the case of the favorite eye and shrink for the opposite one. This condition reveals the existence of a time window during which a cortical selection is operated in the case the visual input from one eye prevails over the other, where visual cortical neurons change their physiological properties preferring the active eye. 

ODP has been extensively studied by means of now-classical approaches ranging from mass physiology studies to the identification of single neuronal properties, as well as approaches probing electrophysiological, behavioral and molecular properties [26]. All the examined aspects have shown profound morphological and functional alterations following manipulations of the visual experience in the cortex of all the species studied, which include monkeys [27], ferrets [28] and rodents [29]. 

Mice lack organized ODCs; however, they have binocular neurons able to integrate inputs from the two eyes. Monocular deprivation in mice induces a shift of ocular dominance of cortical neurons and in visual acuity of the deprived eye, which is markedly reduced. The relatively simple visual system organization of these rodents has allowed revealing the existence of a highly sensitive critical period in visual cortical maturation; this form of plasticity likely constitutes a process evolved to match the left and right eye receptive fields of binocular neurons, ultimately resulting in a particularly well-developed depth perception.

As stated before, the knowledge that brain structures can be modified by experience is old, and well set for the visual system; yet, proving this concept for the human brain experimentally has been possible only with the advent of functional imaging techniques and for animal studies with high-resolution optical methods. Presently, pathological or experience-driven modifications of spine dynamics can be followed in real time by two-photon microscopy [30,31]. However, sometimes plasticity might be hard to disclose. For certain areas of the CNS, plasticity can be simply missing or reduced for strategic reasons, as is the case of the mammalian retina, discussed below.

## 4. The Elusive Retinal Plasticity

Experimental evidence of visual cortical plasticity triggered by dark rearing prompted the search for analogous modifications in neurons located more peripherally than the cortex but still profoundly impacted from light deprivation, primarily the retina, an outpost of the CNS endowed with cells directly sensitive to light. Similarly to cortical neurons receiving an input from the deprived eye, one would expect that photoreceptors and all the downstream neurons contributing to the outer and inner retinal circuitry (bipolar and ganglion cells and the inhibitory interneurons, horizontal and amacrine cells), had to undergo conspicuous changes in synaptic organization, strength and, consequently, in functional properties, following major manipulations of the light input. However, this hypothetical plasticity, which has been searched for, is rather limited, contributing to the fundamental idea of a retina with limited capabilities to undergo long-term modifications in response to a changing environment.

Some degree of plasticity is clearly associated with the late part of retinal development and circuit refinement, as well as with pathological conditions leading to the death of large fractions of neurons, as explained below.

## 5. Plasticity during Retinal Development

A conspicuous part of retinal development takes place after eye opening, which, in most mammals, represents a period of maturation of retinal circuitry and changes of synaptic activity and connectivity. In the mature retina, separate retinal ganglion cell (RGC) types respond to the onset of light (ON responses), to the termination of light (OFF responses) or to both (ON–OFF responses). These functional properties correlate with ganglion cell dendritic stratification in the inner plexiform layer (IPL) of the retina, where dendrites of ON RGCs stratify in the sublamina b (innermost tier) of the IPL, where they receive synaptic inputs from ON cone bipolar cells; OFF RGCs stratify in sublamina a (outermost tier) of the IPL, where they receive input from OFF cone bipolar cells, while ON–OFF RGCs are bistratified, with ramifications of dendrites extended into both ON and OFF sublaminae. During early development, however, immature retinal ganglion cell dendrites ramify diffusely throughout the IPL. The process of their maturation and convergence into monostratified ON and OFF layers is strictly related to visual stimulation after eye opening. 

Classical experiments [32,33] show how light deprivation affects ON versus OFF responsiveness of retinal ganglion cells: in mice born and reared in the dark for 4 weeks, the fraction of ON–OFF RGCs remains abnormally high; their dendrites correspondently continue to maintain a multistratified, immature arrangement in the IPL. Hence, visual-driven activity, even that induced by light passing across closed eyelids [34], is necessary to promote appropriate stratification of RGC dendrites, which, in turn, is a signature of their functional properties. It is visual experience that regulates plastic pruning of RGC dendrites into ON or OFF sublaminae, driving the emergence of pure ON versus OFF responses. 

These experiments explain previous findings demonstrating that acute pharmacological blockade of ON, depolarizing bipolar cells, also impairs the stratification of RGC dendritic trees [35], which is evidently regulated from afferent activity in glutamatergic photoreceptors. 

Additional functional changes in RGC physiology and receptive field properties can be induced by complete light deprivation from birth to past the critical period, resulting in a permanent reduction in the receptive field size and amplitude of the light response. Moreover, ganglion cell receptive fields in visually deprived animals exhibit an anomalous mismatch in the spatial distribution of inhibitory and excitatory afferents, which obviously affects their physiological properties [36]. 

An interesting example of retinal developmental plasticity has been revealed recently in a mouse strain in which one particular type of bipolar cell (named B6), postsynaptic to cones, could be selectively removed. Single-cell electrophysiology demonstrated that ONalpha-RGCs, which normally receive an input from the missing bipolar cell, readjusted their connectivity in such a way to preserve their original properties in terms of contrast and temporal frequency response functions [37]. This rewiring strategy represents a form of homeostatic plasticity and is functionally oriented to stabilize visual information sent to the brain.

In another transgenic mouse, controlled degeneration of 50% cones induced in the young animal highlighted the existence of three types of bipolar cells that could precisely reconstitute their original input synapse numbers, while one type missed this type of homeostatic plasticity [38]. This property was clearly limited to the retinal developmental stage and declined rapidly during maturation. Similarly, in mice in which different fractions of rod bipolar cells (RBCs) could be removed during development, it was observed that dendrites of the remaining RBCs expanded gradually in the outer retina to contact rods, and synaptogenesis was adjusted as a function of rod bipolar cell density to maintain a retinal response in dim light [38]. Thus, as for its core definition, retinal homeostatic plasticity was used here to stabilize activity in developing circuits and demonstrated its cell-type specificity and age-dependence. The relevance of homeostatic plasticity in the CNS in general is highlighted by its impairments in numerous diseases of neural development [39].

Changes observed in RGCs by manipulating their development are an example of plasticity, not dissimilar from the shaping of ocular dominance columns early driven by eye inputs traveling to the visual cortex.

An interesting set of changes in the organization of RGCs can be observed upon exposing laboratory animals to environmental enrichment (EE), which is an experimental paradigm to enhance contact with sensory, motor and social stimulation and a proven tool to increase CNS plasticity. Fetuses carried by rodent mothers exposed to EE during pregnancy show an accelerated migration of neural progenitors in the retina, as well as an anticipated wave of naturally occurring cell death taking place during normal retinal development [40]. These effects are mediated by insulin-like growth factor 1, which reaches the babies through maternal milk. EE also accelerates the development of RGC dendrites and prevents the consequences of dark rearing, counteracting the dropdown in BDNF occurring in the dark and promoting a regular segregation of dendrites of these neurons [41]. 

Altogether, consistent experimental evidence demonstrates that the development of the entire visual system (from the retina to the visual cortex) is highly sensitive to environmental stimuli.

Plastic changes analogous to those occurring in RGCs upon developmental manipulations have also been searched for in neurons located downstream photoreceptors but have been hard to reveal. Recently, some functional consequences of sensory deprivation through developmental dark rearing have been brought to light by studying the physiological properties of rod and cone bipolar cells. Specifically, a net decrement in the synaptic strength of cones and corresponding bipolar cells has emerged in dark-reared mice, while rod bipolar cells, postsynaptic to rods, have been demonstrated to be unaffected [42], even though rod bipolar cells share with ON-cone bipolar cells the same (metabotropic) glutamate receptor, mGluR6. Evidently, the localization of mGluR6 in dendrites of bipolar cells is a light-dependent process only in cone bipolars, and this confers to these cells some plasticity as well as a higher susceptibility to environmental changes. The advantage of a differential bipolar cell sensitivity toward the environment is not yet clear; yet, considering that the cone system is evolutionary more antique than the rod system [43], it is possible to speculate that the cone circuitry of the mammalian retina had a longer time for diversification and further refinement.

Synaptic connections engaged between cones and rod bipolar cells are rare in a normal mammalian retina, yet experiments with genetically altered mice show that the developing retina is plastic enough to break this rule. In the retina of neural retina leucine zipper (Nrl) knock-out mice, neuroblastic precursors destined to differentiate into photoreceptors miss the fundamental transcription factor that instructs them to become rods, which therefore do not form at all. In a retina full of supernumerary cones, rod bipolar cells continue to show their fundamental morphological traits and pattern of lamination but form synaptic connections with cones, showing that their connectivity commitment is not absolute [44]. The ability to establish connections involving neurons normally not linked to each other demonstrates a type of plasticity probably tuned to guarantee vision, capable of recruiting partners that otherwise would not be connected at all, at the expense of communication. 

Similarly, a mouse expressing the antiapoptotic gene bcl-2 and undergoing a limited rate of natural cell death during development displays a brain and retina with much higher neuronal numbers than normal and with completely altered quantitative relations among synaptic partners. The various populations of retinal cells (i.e., bipolar cells, ganglion cells) are not covaried; for instance, there are proportionally many more rod bipolar cells and ganglion cells than cholinergic amacrine cells, while the population of dopaminergic amacrines is disproportionately large [45]. It is somewhat amazing that, in this uncommon retina, fundamental circuits are maintained and vision is preserved [46] despite major alterations in circuitry balance.

Altogether, alterations induced in the developing retina by abnormal experience or genetic manipulations demonstrate the existence of a degree of homeostatic plasticity analogous to that of the visual cortex [47], with ganglion cells more easily showing plastic changes compared to neurons of the outer retina. Such a “conservative reactivity” can be considered as a tool to preserve vision even when expected synaptic partners are abnormal in identity or number.

## 6. Retinal Regressive Remodeling in Disease

Numerous genetic diseases affect the physiology and survival of photoreceptors, leading to progressive degeneration and death of these cells. In typical retinitis pigmentosa (RP), mutations in more than 60 identified genes with disparate functions all cause a rod–cone degeneration resulting in blindness. Various laboratories (including ours) have investigated the consequences of the progressive demise of the largest neuronal population in the retina upon the remaining neurons, which constitute a biological platform that can be used for restoration therapies. Promising approaches relying on the inner retina exploit cell transplantation, implantation of an electronic prosthesis and optogenetic replacement of vision [48]. In RP and similar diseases, all second-order neurons (i.e., rod bipolar, cone bipolar and horizontal cells) sooner or later become deafferented, as photoreceptors die out at different rates, dictated by the causative mutation. Ganglion cells, the last neurons of the retinal vertical pathway, although not connected to photoreceptors directly, are also functionally deafferented and deprived of light-initiated inputs. In most human cases, the RP phenotype manifests in late adolescence, well outside the timeframe of visual system development. Rodent models are usually selected to better mimic human mutations and genotype–phenotype correlations, and strains undergoing retinal degeneration past development are preferred. Hence, retinal plasticity in RP, if any, should not be expected to be the same following dark rearing or monocular deprivation experiments, nor like that arising because of genetic manipulations becoming effective at developmental stages of CNS specification, as is the case for Nrl ablation or bcl-2 overexpression. 

Rather than plasticity, indeed, every process causing photoreceptor loss concomitantly triggers a series of events collectively known as retinal remodeling, a progression of alterations similar to maladaptive changes common to other CNS neurodegenerative diseases and believed to limit rescue approaches of all kinds [49,50,51].

Pioneering work [52] on animal models of RP and postmortem retinal samples from RP donors revealed abnormal changes occurring in the inner retina as a consequence of photoreceptor loss: these included profuse sprouting of surviving rods and of GABA-ergic amacrine and horizontal cells and formation of aberrant neurites of different sizes and orientations reaching the surface of Müller cells in the inner retina. Like the process of formation of a glial scar occurring in other forms of CNS neurodegeneration, hypertrophy and migration of macroglia (Müller cells and astrocytes) contributed to the formation of glial seals in the outer and inner retina, while the number of inner retinal neurons (bipolar cells, ganglion cells, etc.) gradually declined. Various studies revealed the general occurrence of extensive and progressive disease-driven retinal remodeling, initially invisible to routine histological methods, contributing to the general concept of a progressive deterioration of retinal structure and function, likely representing an obstacle for vision restoration approaches. 

Some categorized phases of remodeling are now known for RP, and the main steps are clearly secondary to rod and cone death; they usually display the same spatial distribution (topography) of photoreceptor loss, namely a periphery-to-center gradient for human RP and a reversed, center-to-periphery, gradient in the rodent models of the disease. Remodeling affects first and more intensely cells directly connected to rods and cones and is attenuated in time and space, moving radially from photoreceptors toward ganglion cells. Some aspects of retinal remodeling (including cell death, change in morphology and dendritic loss) have been described by microscopy techniques based on the use of cell-type-specific antibody staining, while more detailed changes in neuronal number have required extensive cell counting and population studies [53,54,55,56,57]. 

**Figure 1:**
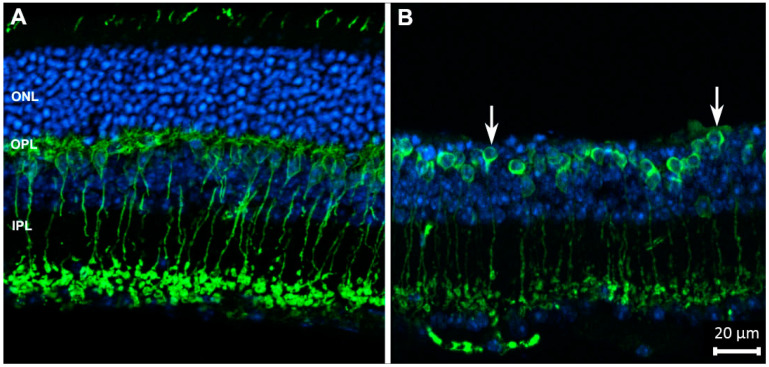
Rod bipolar cells (in green) of wild-type (**A**) and degenerating retinas (**B**), stained with antibodies against PKCα. Blue: nuclear counterstaining. Upon photoreceptor degeneration, the outer nuclear layer (ONL) becomes thinner and dendritic arbors of bipolar cells progressively retract from the outer plexiform layer (OPL) (arrows), while the complexity of their axonal arbors in the inner plexiform layer (IPL) decreases. These are typical changes occurring in neurons as a reaction to deafferentation. Abbreviations are the same in the following figures.

**Figure 2:**
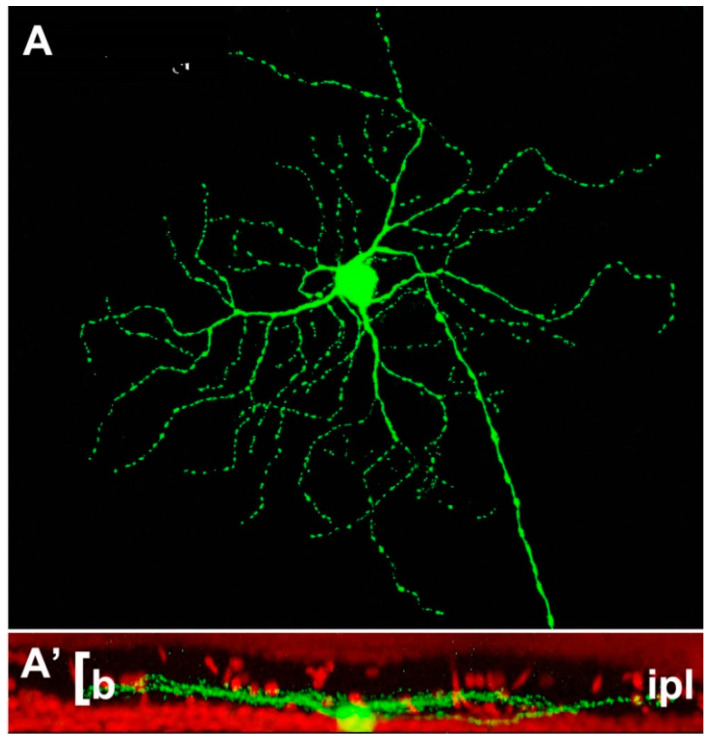
Example of retinal ganglion cell (**A**) from a Thy-1-GFP expressing mouse strain. The cell shows a narrow pattern of dendritic stratification in sublamina b (the innermost tier) of the IPL (**A’**). This pattern is not altered by the rd1 mutation hosted by this strain, one of the most aggressive genetic defects, leading to a rapid degeneration of photoreceptors in less than two weeks of postnatal life (Damiani et al., 2009).

**Figure 3:**
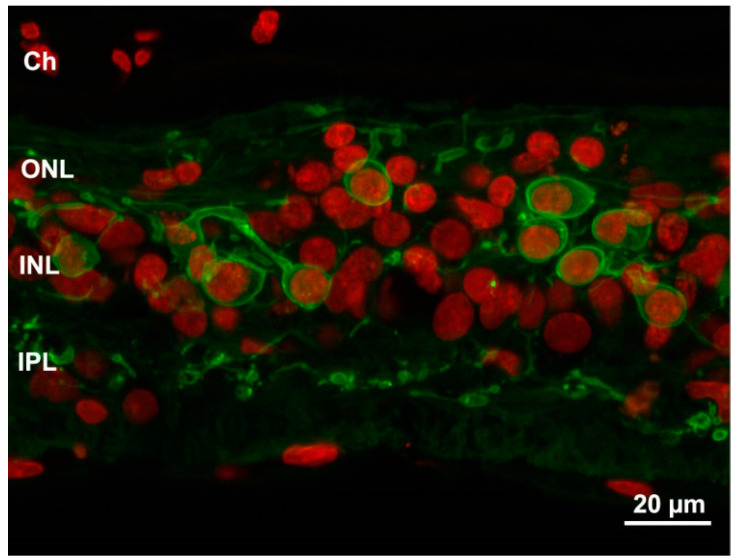
Profound remodeling of human retina shown in a sample from an advanced RP stage. Green: PKC antibody staining of bipolar cells. Red: Nuclear counterstaining. The typical laminar structure of the retina is completely lost and both dendritic and axonal arbors of bipolar cells are highly retracted and irregular. Ch: choroid.

Some aspects of retinal remodeling are not obvious and so subtle to need specific tools allowing to associate molecular changes to individual cell types. Computational molecular phenotyping (CMP) combines immunocytochemistry with cell-type-specific antibodies (usually directed against specific metabolites), high-resolution microscopy on thin sections and computational analysis. This method has been implemented and used extensively by the laboratories of Robert Marc and Bryan Jones to reveal profound changes in the metabolic signature of various retinal cells in different forms of degenerative diseases, predictive of parallel dysfunctions [58]. These authors have distinguished in retinal remodeling three phases, the first characterized by photoreceptor stress and early rearrangements; the second involving all the main glial types, microglia and Müller cells, as well as the retinal pigment epithelium (RPE); and the third being when abortive attempts of neuritic formation and ectopic rewiring are made. From this stage on, the effects of neuronal death are quite massive, and the retina becomes restructured, visibly very different from the normal, highly organized, tissue [59]. Examples of steps in remodeling can be observed in Figure 1, Figure 2 and Figure 3.

Noticeably, ganglion cells, the only exit neurons from the retina, appear inherently stable: not only is their survival rate quite high (and this constitutes a favorable condition for repair strategies), but their dendritic arborizations change very little following extensive photoreceptor loss, unlike other neurons of the CNS undergoing similar deafferentation processes. Their main features (layer of lamination, size, branching pattern) are minimally affected, so cells can be still categorized as ON, OFF or ON–OFF based on the pattern of dendritic stratification in the IPL [60,61] (Figure 1, Figure 2). 

Yet, electrophysiological studies in retinas with photoreceptor degeneration have demonstrated spontaneous (paroxysmal) spiking activity in ganglion cells, even when sensory input was almost completely absent [62]. These initial studies have been confirmed in various retinal degeneration mutants with the demonstration that the spontaneous activity recorded from ganglion cells arises from aberrant rewiring of processes in the plexiform layers [63,64]. Specifically, a well-proven component of RGC spontaneous discharge originates in the rod–cone amacrine known as AII and its network with ON cone bipolar cells, which comprises glycinergic as well as glutamatergic synapses and in which cells are joined by homotypic and heterotypic gap junctions. The latter seem intrinsically capable to generate the oscillations typical of retinal degenerations.

The spontaneous, oscillatory hyperactivity of a degenerating retina is usually considered maladaptive; this idea is supported by the finding that its pharmacological inhibition leads to an improvement of the outcome of prosthetic stimulation designed for vision restoration [65]. Yet, it cannot be excluded that spontaneous activity leads some biological benefit to ganglion cells, possibly conferring these neurons increased capability of survival, as demonstrated by experiments conducted on ganglion cells in vivo and in vitro [66]. Electrical activity is fundamental for neurons in general, supporting some crucial functions, such as the uptake and release of growth factors. Ganglion cells, whose axons form the only retinofugal pathway, represent the last frontier exploitable to restore visual signals and send them to the brain. 

Ganglion cell viability and survival in retinal degeneration are crucial for reasons going beyond the possibility to regain light sensitivity: some ganglion cells, containing melanopsin, connect to the central clock of the suprachiasmatic nucleus, regulating circadian rhythms which, in turn, influence fundamental biological processes, such as reproductive activities, sensitivity to seasonal changes and migration. These ancestral biological processes, depending upon ganglion cell input, make the preservation of these neurons highly relevant for the species.

A constant feature of remodeling is represented by the process of reactive gliosis, by which Müller cells enlarge and form a glial seal, which is generally considered almost impenetrable and fills the spaces left empty by the dead cells. Müller cell hyper-reactivity and upregulation of GFAP are common to various forms of retinal pathological conditions [67]. Although less well known, a process of hypertrophy and hyper-reactivity is also followed by astrocytes, which occupy the optic fiber layer; though anatomically distant from degenerating photoreceptors, astrocytes are early sensors of any local change in the extracellular milieu and tissue metabolism due to their participation to the neurovascular unit [68]. 

Recently, we observed previously undetected remodeling in the RPE as well, where changes parallel the well-known process of melanin migration described in animal models of RP and human cases. The newly detected changes rather mimic alterations of the RPE described in macular degeneration [69]. 

**Figure 4:**
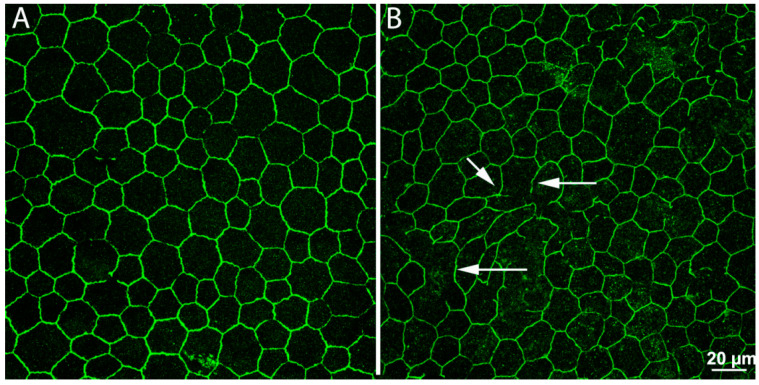
Remodeling in the retinal pigment epithelium (RPE). Whole-mount preparations of RPE leaflets stained with antibodies against ZO-1, revealing junctional complexes between cells. In the preparation from a normal, wild-type mouse (**A**), the arrangement of ZO-1 positive elements is regular and continuous. In a retinal degeneration mouse (**B**), regularity and continuity have been lost. Numerous holes (arrows) are visible, making the outer blood–retina barrier pathologically leaky.

We found that, in various animal models of RP, RPE cells undergo a process of progressive interruption of the array of tight junctions joining their apical sides, which are fundamental for the maintenance of the outer blood–retina barrier. These junctions become leaky, allowing the free entry of high-molecular-weight species into the retina and revealing a vulnerability of a fundamental defense system (Figure 4). It is not yet known if other cell types are involved in this process (i.e., cells of the immune system, occupying the outer side of the RPE and known to have phagocytic activity), yet a breakdown of the RPE is believed to contribute to RP progression and further demise of photoreceptors. Alterations in the RPE reinforce the notion that remodeling following photoreceptor loss is a global process, involving every neural and non-neural component of the retina.

Despite tremendous genetic heterogeneity of RP, animal and human studies show that regressive events occurring in the inner retina are somewhat stereotyped and predictable, making them presumably easier to control with therapeutic approaches, provided that large clinical differences among RP individuals do not challenge present findings.

In a recent study, we analyzed the fine structure of the retina of the Tvrm4 rhodopsin mutant mouse, which undergoes a fast degeneration of photoreceptors when exposed for a few minutes to a source of very bright light. The nonexposed, peripheral retina is virtually undistinguishable from that of a nonmutant, wild-type mouse, and the model can be exploited to evaluate the entity of retinal remodeling in the young adult, closely mimicking the typical age of RP phenotype manifestation in humans [70]. Despite bipolar cell dendritic retraction and moderate loss of horizontal cells, the survival rate of various cell types is very high, ensuring an overall preservation of the inner retina. A significant ultrastructural and numerical maintenance of conventional synapses and gap junctions in the inner plexiform layer is also observed [71]. Indeed, the number of synaptic ribbons, which in the inner retina are contained in glutamatergic terminals of bipolar cells, gradually declines, while their ultrastructure becomes transiently abnormal. Still, the overall circuitry involving bipolar cells appears maintained despite the complete loss of photoreceptors. These observations hold true even 2 months past light exposure, a time corresponding to a medium stage of advancement of the disease. Studies on this mutant show that although not plastic, the mature retina has a larger than expected potential to maintain viability of key neurons (i.e., bipolar cells) used to transfer information along the “vertical” pathway, even when photoreceptor loss is complete and yet a fraction of adjacent retina is still intact. Obviously, this constitutes a promising finding for the success of vision restoration strategies implemented when at least some retinal patches are still viable. 

In conclusion, remodeling of the retina in RP is very different from the classical hippocampal ability to rearrange in a positive sense, the most relevant retinal feature being a capability to resist major, secondary remodeling during the initial phases following photoreceptor death. 

Whether homeostatic, restorative plasticity does occur during photoreceptor degenerative disease to help maintain some vision is not really known. Recent studies in an RP mouse carrying the P23H mutation in rhodopsin demonstrate modulation of the retinal transcriptomic network, with the acquisition of a molecular signature reminiscent of a neurodevelopmental state, and a potentiation of rod–rod bipolar cell signaling [72]. This correlates to an unexpected high sensitivity of night vision despite a loss of about 50% of rods, suggesting the occurrence of a true compensatory, plastic activity of the retinal circuitry, acting to maintain vision.

## 7. Some Room for Adaptive Plasticity in the Retina

Recent studies support the possibility of the existence of additional adaptive, rather than maladaptive, retinal plasticity. Ground squirrels, which possess an all-cone retina, have dedicated cone bipolar cells, called S-cone bipolars, establishing individual synaptic connections with cones. This one-to-one arrangement is strongly reminiscent of cone-to-bipolar private connections occurring in the primate fovea. Noticeably, the experimental photoablation of cones in the mature retina of these squirrels leads to the successful regrowth of bipolar cell dendrites, which extend in the damaged part of the retina and establish new connections with surviving cones. The process has led to defining the sprouting bipolar cell as a neuron which “remembers” its synapse [73]. To be noted, in ground squirrels, connections between cones and homologous bipolar cells undergo remodeling during hibernation, when all the presynaptic machinery is decomposed, to be reassembled at the time of animal awakening [74]. Functional recovery of visual responses follows quickly, according to a well-organized and rapid process [75]. Possibly, in photoablation conditions the memory of these seasonal events helps bipolar cells to adjust to a pathological situation, ensuring vision preservation.

Similar studies on the mature mouse retina treated with sectorial photoablation of photoreceptors reveal that rod or cone bipolar cells can regrow dendrites and form new synapses with their correct targets (i.e., rods and cones) also outside of their normal dendritic field, in what appears as an attempt to compensate for the dropdown in photoreceptor number and for maintaining retinal output close to normal levels [76]. This plasticity can be considered homeostatic; although its final impact on retinal physiology has yet to be understood, it should be carefully considered in designing treatment strategies for blinding diseases, especially when choosing the timing of intervention. 

A peculiar form of adaptive plasticity has been described for photoreceptor synapses of zebrafish, where synaptic ribbons undergo dramatic diurnal alterations, being very prominent during the day but disappearing almost entirely at night [77]. Since larval zebrafish almost entirely lose their ability to respond to light at night, the disassembly of ribbon synapse (a highly energy-demanding piece of machinery) might be part of an adaptive process for larvae to conserve energy. This mechanism seems a form of intermediate biological process between hemostatic plasticity and adaptation.

The ability of adult mouse cone bipolar cells to undergo a compensatory dendritic regrowth (illustrated above) appears anyhow a circumscribed event when compared to the capacity of the retina of cold-blooded vertebrates to fully regenerate, by virtue of the neurogenetic power of multiple cellular sources, namely retinal stem cells in the circumferential marginal zone (CMZ), the RPE and Müller glia, a true source of retinal regeneration [78]. The long-known fact that in teleost fish and amphibians the retina and the eye continue to grow throughout the animal’s life [79] has promoted studies clarifying the mechanisms through which cold-blooded Müller cells can put into action a program of cell genesis in which derived cells differentiate into retinal neurons, ensuring a robust form of retinal repair. These studies have prompted the search for molecules and pathways involved in neuronal plasticity and repair in species in which these processes are still very active [80], in perspective understanding how retinal neurogenic potential is suppressed in mammalians and learning how it can be unlocked to promote retinal repair. 

Given the fact that cortical plasticity is active, true effects of retinal compensatory mechanisms on retinal degeneration models and on patients are hard to evaluate. Cortical compensation could explain the behavioral ability of RP individuals to adjust for long times to the progressive decrement in rods and their effective usage of residual cone-mediated vision until an advanced disease state. Similarly, the delay in vision loss observed in retinal degeneration mice exposed to environmental enrichment [81] could be partially ascribed to plastic responses occurring in the cortex, rather than in the retina alone. Cortical “spared plasticity” could be higher than expected even in adult subjects and should be exploited to burst residual retinal plasticity to favor repair therapies [82,83].

## 8. Concluding Remarks

In comparing the substantial plasticity of the hippocampus and cortex to their limited counterpart in the retina, one should consider that peculiarity of the retina is that it is organized like the cortex, but it also contains the primary sensory neurons. The proximity of the sensors to the primary nervous center where a considerable amount of processing takes place confers advantages (among others, great sensitivity and speed) but also poses design constraints. If plastic rearrangements of retinal circuitry were the rule, this would probably implicate undesired variability in the transmission of important sensorial information and consequent risk of message infidelity. Sensors should ensure sensitivity and fidelity, and the downstream circuitry is expected to ensure stability. Indeed, the incredible sensorial adaptation typical of the visual system, which can operate over a range of light levels spanning approximately 10 logarithmic units, is obtained through the high amplification proper of the phototransduction cascade, a high speed of communication of glutamatergic synapses endowed with specialized ribbon machinery and a dedicated wiring diagram with high convergence and intrinsic adaptation abilities. Probably, all this limits the possibilities of a true, cortical-like, plasticity.

Injured cortical neurons, in a favorable environment, can revert their transcriptional profile to that of embryonic cortical blasts and regrow axons, suggesting that the genetic program for regrowth (and plasticity) is not irremediably lost in mature neurons but, more probably, silenced. For retinal cells, this program is likely maintained locked for safety reasons. A whole CNS undergoing continuous renovation would be metabolically expensive at the cost of efficiency: retinal plasticity, rather than being completely gone, can be more adequately believed to be held in place actively by efficient and safe brake mechanisms [84] that guarantee the fidelity of vision, the dominant sensory modality for the human species. It cannot be excluded that, in the future, dormant retinal plasticity can be unlocked for therapeutic purposes.

## References

[1] Berlucchi G., Buchtel H.A. (2009). Neuronal plasticity: Historical roots and evolution of meaning. Exp. Brain Res..

[2] Bliss T.V., Lomo T. (1973). Long-lasting potentiation of synaptic transmission in the dentate area of the anaesthetized rabbit following stimulation of the perforant path. J. Physiol..

[3] Kandel E.R., Dudai Y., Mayford M.R. (2014). The molecular and systems biology of memory. Cell.

[4] Levy W.B., Steward O. (1983). Temporal contiguity requirements for long-term associative potentiation/depression in the hippocampus. Neuroscience.

[5] Magee J.C., Grienberger C. (2020). Synaptic Plasticity Forms and Functions. Annu. Rev. Neurosci..

[6] Malenka R.C., Bear M.F. (2004). LTP and LTD: An embarrassment of riches. Neuron.

[7] Zenke F., Gerstner W. (2017). Hebbian plasticity requires compensatory processes on multiple timescales. Philos. Trans. R. Soc. B. Biol. Sci..

[8] Lamprecht R., le Doux J. (2004). Structural plasticity and memory. Nat. Rev. Neurosci..

[9] Soto M., Cai W., Konishi M., Kahn C.R. (2019). Insulin signaling in the hippocampus and amygdala regulates metabolism and neurobehavior. Proc. Natl. Acad. Sci. USA.

[10] Cooper D.D., Frenguelli B.G. (2021). The influence of sensory experience on the glutamatergic synapse. Neuropharmacology.

[11] Harris K.M. (2020). Structural LTP: From synaptogenesis to regulated synapse enlargement and clustering. Curr. Opin. Neurobiol..

[12] Ma S., Zuo Y. (2021). Synaptic modifications in learning and memory—A dendritic spine story. Semin. Cell Dev. Biol..

[13] Sorrells S.F., Paredes M.F., Cebrian-Silla A., Sandoval K., Qi D., Kelley K.W., James D., Mayer S., Chang J., Auguste K.I. (2018). Human hippocampal neurogenesis drops sharply in children to undetectable levels in adults. Nature.

[14] Aimone J.B., Li Y., Lee S.W., Clemenson G.D., Deng W., Gage F.H. (2014). Regulation and function of adult neurogenesis: From genes to cognition. Physiol. Rev..

[15] Kumar A., Pareek V., Faiq M.A., Ghosh S.K., Kumari C. (2019). ADULT NEUROGENESIS IN HUMANS: A Review of Basic Concepts, History, Current Research, and Clinical Implications. Innov. Clin. Neurosci..

[16] Beckmann D., Feldmann M., Shchyglo O., Manahan-Vaughan D. (2020). Hippocampal Synaptic Plasticity, Spatial Memory, and Neurotransmitter Receptor Expression Are Profoundly Altered by Gradual Loss of Hearing Ability. Cereb. Cortex.

[17] Berardi N., Pizzorusso T., Maffei L. (2000). Critical periods during sensory development. Curr. Opin. Neurobiol..

[18] Reh R.K., Dias B.G., Nelson C.A., Kaufer D., Werker J.F., Kolb B., Levine J.D., Hensch T.K. (2020). Critical period regulation across multiple timescales. Proc. Natl. Acad. Sci. USA.

[19] Hubel D.H., Wiesel T.N., le Vay S. (1977). Plasticity of ocular dominance columns in monkey striate cortex. Philos. Trans. R. Soc. B Biol. Sci..

[20] Levelt C.N., Hubener M. (2012). Critical-period plasticity in the visual cortex. Annu. Rev. Neurosci..

[21] Galli L., Maffei L. (1988). Spontaneous impulse activity of rat retinal ganglion cells in prenatal life. Science.

[22] Ackman J.B., Burbridge T.J., Crair M.C. (2012). Retinal waves coordinate patterned activity throughout the developing visual system. Nature.

[23] Stellwagen D., Shatz C.J. (2002). An instructive role for retinal waves in the development of retinogeniculate connectivity. Neuron.

[24] Huberman A.D., Speer C.M., Chapman B. (2006). Spontaneous retinal activity mediates development of ocular dominance columns and binocular receptive fields in v1. Neuron.

[25] Stryker M.P., Harris W.A. (1986). Binocular impulse blockade prevents the formation of ocular dominance columns in cat visual cortex. J. Neurosci..

[26] Hooks B.M., Chen C. (2020). Circuitry Underlying Experience-Dependent Plasticity in the Mouse Visual System. Neuron.

[27] Feller M.B., Scanziani M. (2005). A precritical period for plasticity in visual cortex. Curr. Opin. Neurobiol..

[28] Issa N.P., Trachtenberg J.T., Chapman B., Zahs K.R., Stryker M.P. (1999). The critical period for ocular dominance plasticity in the Ferret’s visual cortex. J. Neurosci..

[29] Huang Z.J., Kirkwood A., Pizzorusso T., Porciatti V., Morales B., Bear M.F., Maffei L., Tonegawa S. (1999). BDNF regulates the maturation of inhibition and the critical period of plasticity in mouse visual cortex. Cell.

[30] Kasai H., Ziv N.E., Okazaki H., Yagishita S., Toyoizumi T. (2021). Spine dynamics in the brain, mental disorders and artificial neural networks. Nat. Rev. Neurosci..

[31] Landi S., Putignano E., Boggio E.M., Giustetto M., Pizzorusso T., Ratto G.M. (2011). The short-time structural plasticity of dendritic spines is altered in a model of Rett syndrome. Sci. Rep..

[32] Tian N., Copenhagen D.R. (2001). Visual deprivation alters development of synaptic function in inner retina after eye opening. Neuron.

[33] Tian N., Copenhagen D.R. (2003). Visual stimulation is required for refinement of ON and OFF pathways in postnatal retina. Neuron.

[34] Feller M.B. (2003). Visual system plasticity begins in the retina. Neuron.

[35] Bodnarenko S.R., Chalupa L.M. (1993). Stratification of ON and OFF ganglion cell dendrites depends on glutamate-mediated afferent activity in the developing retina. Nature.

[36] Di Marco S., Nguyen V.A., Bisti S., Protti D.A. (2009). Permanent functional reorganization of retinal circuits induced by early long-term visual deprivation. J. Neurosci..

[37] Tien N.W., Soto F., Kerschensteiner D. (2017). Homeostatic Plasticity Shapes Cell-Type-Specific Wiring in the Retina. Neuron.

[38] Johnson R.E., Tien N.W., Shen N., Pearson J.T., Soto F., Kerschensteiner D. (2017). Homeostatic plasticity shapes the visual system’s first synapse. Nat. Commun..

[39] Wondolowski J., Dickman D. (2013). Emerging links between homeostatic synaptic plasticity and neurological disease. Front. Cell. Neurosci..

[40] Sale A., Cenni M.C., Ciucci F., Putignano E., Chierzi S., Maffei L. (2007). Maternal enrichment during pregnancy accelerates retinal development of the fetus. PLoS ONE.

[41] Landi S., Sale A., Berardi N., Viegi A., Maffei L., Cenni M.C. (2007). Retinal functional development is sensitive to environmental enrichment: A role for BDNF. Faseb J..

[42] Dunn F.A., della Santina L., Parker E.D., Wong R.O. (2013). Sensory experience shapes the development of the visual system′s first synapse. Neuron.

[43] Morshedian A., Fain G.L. (2017). The evolution of rod photoreceptors. Philos. Trans. R. Soc. Lond. B Biol. Sci..

[44] Strettoi E., Mears A.J., Swaroop A. (2004). Recruitment of the rod pathway by cones in the absence of rods. J. Neurosci..

[45] Strettoi E., Volpini M. (2002). Retinal organization in the bcl-2-overexpressing transgenic mouse. J. Comp. Neurol..

[46] Porciatti V., Pizzorusso T., Maffei L. (1999). Vision in mice with neuronal redundancy due to inhibition of developmental cell death. Vis. Neurosci..

[47] Turrigiano G. (2012). Homeostatic synaptic plasticity: Local and global mechanisms for stabilizing neuronal function. Cold Spring Harb. Perspect. Biol..

[48] Zhang Q. (2016). Retinitis Pigmentosa: Progress and Perspective. Asia-Pac. J. Ophthalmol..

[49] Jones B.W., Kondo M., Terasaki H., Lin Y., McCall M., Marc R.E. (2012). Retinal remodeling. Jpn. J. Ophthalmol..

[50] Jones B.W., Pfeiffer R.L., Ferrell W.D., Watt C.B., Marmor M., Marc R.E. (2016). Retinal remodeling in human retinitis pigmentosa. Exp. Eye Res..

[51] Marc R.E., Jones B.W., Watt C.B., Strettoi E. (2003). Neural remodeling in retinal degeneration. Prog. Retin. Eye Res..

[52] Fariss R.N., Li Z.Y., Milam A.H. (2000). Abnormalities in rod photoreceptors, amacrine cells, and horizontal cells in human retinas with retinitis pigmentosa. Am. J. Ophthalmol..

[53] Strettoi E., Pignatelli V., Rossi C., Porciatti V., Falsini B. (2003). Remodeling of second-order neurons in the retina of rd/rd mutant mice. Vision Res..

[54] Pignatelli V., Cepko C.L., Strettoi E. (2004). Inner retinal abnormalities in a mouse model of Leber’s congenital amaurosis. J. Comp. Neurol..

[55] Gargini C., Terzibasi E., Mazzoni F., Strettoi E. (2007). Retinal organization in the retinal degeneration 10 (rd10) mutant mouse: A morphological and ERG study. J. Comp. Neurol..

[56] Lin B., Masland R.H., Strettoi E. (2009). Remodeling of Cone Photoreceptor Cells After Rod Degeneration in Rd Mice. ARVO Meet. Abstr..

[57] Strettoi E. (2015). A Survey of Retinal Remodeling. Front. Cell Neurosci..

[58] Marc R.E., Jones B.W., Anderson J.R., Kinard K., Marshak D.W., Wilson J.H., Wensel T., Lucas R.J. (2007). Neural reprogramming in retinal degeneration. Investig. Ophthalmol. Vis. Sci..

[59] Pfeiffer R.L., Marc R.E., Jones B.W. (2020). Persistent remodeling and neurodegeneration in late-stage retinal degeneration. Prog. Retin. Eye Res..

[60] Damiani D., Novelli E., Mazzoni F., Strettoi E. (2012). Undersized dendritic arborizations in retinal ganglion cells of the rd1 mutant mouse: A paradigm of early onset photoreceptor degeneration. J. Comp. Neurol..

[61] Mazzoni F., Novelli E., Strettoi E. (2008). Retinal ganglion cells survive and maintain normal dendritic morphology in a mouse model of inherited photoreceptor degeneration. J. Neurosci..

[62] Stasheff S.F. (2008). Emergence of sustained spontaneous hyperactivity and temporary preservation of OFF responses in ganglion cells of the retinal degeneration (rd1) mouse. J. Neurophysiol..

[63] Euler T., Schubert T. (2015). Multiple Independent Oscillatory Networks in the Degenerating Retina. Front. Cell. Neurosci..

[64] Haq W., Arango-Gonzalez B., Zrenner E., Euler T., Schubert T. (2014). Synaptic remodeling generates synchronous oscillations in the degenerated outer mouse retina. Front. Neural. Circuit..

[65] Barrett J.M., Degenaar P., Sernagor E. (2015). Blockade of pathological retinal ganglion cell hyperactivity improves optogenetically evoked light responses in rd1 mice. Front. Cell. Neurosci..

[66] Corredor R.G., Goldberg J.L. (2009). Electrical activi.ity enhances neuronal survival and regeneration. J. Neural. Eng..

[67] Sethi C.S., Lewis G.P., Fisher S.K., Leitner W.P., Mann D.L., Luthert P.J., Charteris D.G. (2005). Glial remodeling and neural plasticity in human retinal detachment with proliferative vitreoretinopathy. Investig. Ophthalmol. Vis. Sci..

[68] Luna G., Keeley P.W., Reese B.E., Linberg K.A., Lewis G.P., Fisher S.K. (2016). Astrocyte structural reactivity and plasticity in models of retinal detachment. Exp. Eye Res..

[69] Napoli D., Biagioni M., Billeri F., di Marco B., Orsini N., Novelli E., Strettoi E. (2021). Retinal Pigment Epithelium Remodeling in Mouse Models of Retinitis Pigmentosa. Int. J. Mol. Sci..

[70] Gargini C., Novelli E., Piano I., Biagioni M., Strettoi E. (2017). Pattern of retinal morphological and functional decay in a light-inducible, rhodopsin mutant mouse. Sci. Rep..

[71] Stefanov A., Novelli E., Strettoi E. (2020). Inner retinal preservation in the photoinducible I307N rhodopsin mutant mouse, a model of autosomal dominant retinitis pigmentosa. J. Comp. Neurol..

[72] Leinonen H., Pham N.C., Boyd T., Santoso J., Palczewski K., Vinberg F. (2020). Homeostatic plasticity in the retina is associated with maintenance of night vision during retinal degenerative disease. eLife.

[73] Beier C., Palanker D., Sher A. (2018). Stereotyped Synaptic Connectivity Is Restored during Circuit Repair in the Adult Mammalian Retina. Curr. Biol..

[74] Merriman D.K., Sajdak B.S., Li W., Jones B.W. (2016). Seasonal and post-trauma remodeling in cone-dominant ground squirrel retina. Exp. Eye Res..

[75] Zhang H., Sajdak B.S., Merriman D.K., McCall M.A., Carroll J., Lipinski D.M. (2020). Electroretinogram of the Cone-Dominant Thirteen-Lined Ground Squirrel during Euthermia and Hibernation in Comparison with the Rod-Dominant Brown Norway Rat. Investig. Ophthalmol. Vis. Sci..

[76] Care R.A., Kastner D.B., de la Huerta I., Pan S., Khoche A., Della Santina L., Gamlin C., Santo Tomas C., Ngo J., Chen A. (2019). Partial Cone Loss Triggers Synapse-Specific Remodeling and Spatial Receptive Field Rearrangements in a Mature Retinal Circuit. Cell. Rep..

[77] Emran F., Rihel J., Adolph A.R., Dowling J.E. (2010). Zebrafish larvae lose vision at night. Proc. Natl. Acad. Sci. USA.

[78] Gallina D., Todd L., Fischer A.J. (2014). A comparative analysis of Muller glia-mediated regeneration in the vertebrate retina. Exp. Eye Res..

[79] Reh T.A., Levine E.M. (1998). Multipotential stem cells and progenitors in the vertebrate retina. J. Neurobiol..

[80] Palazzo I., Deistler K., Hoang T.V., Blackshaw S., Fischer A.J. (2020). NF-kappaB signaling regulates the formation of proliferating Muller glia-derived progenitor cells in the avian retina. Development.

[81] Guadagni V., Biagioni M., Novelli E., Aretini P., Mazzanti C.M., Strettoi E. (2019). Rescuing cones and daylight vision in retinitis pigmentosa mice. FASEB J..

[82] Baroncelli L., Lunghi C. (2021). Neuroplasticity of the visual cortex: In sickness and in health. Exp. Neurol..

[83] Begenisic T., Mazziotti R., Sagona G., Lupori L., Sale A., Galli L., Baroncelli L. (2020). Preservation of Visual Cortex Plasticity in Retinitis Pigmentosa. Neuroscience.

[84] Lu Y., Brommer B., Tian X., Krishnan A., Meer M., Wang C., Vera D.L., Zeng Q., Yu D., Bonkowski M. (2020). Reprogramming to recover youthful epigenetic information and restore vision. Nature.

